# Prognostic Values of Inflammatory Indexes and Clinical Factors in Patients with Epidermal Growth Factor Receptor Mutations in Lung Adenocarcinoma and Treated with Tyrosine Kinase Inhibitors

**DOI:** 10.3390/jpm12030404

**Published:** 2022-03-05

**Authors:** Bee-Song Chang, Tai-Chu Peng, Yi-Feng Wu, Tsung-Cheng Hsieh, Chun-Hou Huang

**Affiliations:** 1 Department of Thoracic Surgery, Hualien Tzu Chi Hospital, Buddhist Tzu Chi Medical Foundation, Hualien 970473, Taiwan; r122336@tzuchi.com.tw; 2 School of Medicine, College of Medicine, Tzu Chi University, Hualien 970374, Taiwan; wuyifeng43@tzuchi.com.tw; 3 Department of Nursing, Tzu Chi University, Hualien 970374, Taiwan; ptc2008@gms.tcu.edu.tw; 4 Department of Hematology and Oncology, Hualien Tzu Chi Hospital, Buddhist Tzu Chi Medical Foundation, Hualien 970473, Taiwan; 5 Institute of Medical Sciences, Tzu Chi University, Hualien 970374, Taiwan; tchsieh@gms.tcu.edu.tw

**Keywords:** lung adenocarcinoma, tyrosine kinase inhibitor, epidermal growth factor receptor, treatment-related toxicity, hypoalbuminemia, inflammatory index

## Abstract

This study aimed to access the predictive value of inflammatory indices and clinical factors in toxicity and survival in patients with epidermal growth factor receptor (EGFR)-mutated lung adenocarcinoma receiving first-line tyrosine kinase inhibitor (TKI)-treatment. A total of 259 patients with stage IIIB–IV lung adenocarcinoma and actionable EGFR mutation who received first-line TKI treatment between 2008 and 2020 were retrospectively enrolled and analyzed. The prognostic factors of TKI-related toxicity, overall survival (OS), and progression-free survival (PFS) were identified by using logistic regression analysis and Cox proportional hazards models. Pre-TKI high platelet-to-lymphocyte ratio (PLR) was associated with post-TKI anemia. Hypoalbuminemia was associated with acneiform rash. Elderly age (≥70 years) and lower body mass index (<18.5 kg/m^2^) were also associated with hypoalbuminemia. Elderly age, stage IV, EGFR-mutated with L858R and uncommon mutations, and neutrophil-to-lymphocyte ratio were found to be independent prognostic factors for PFS, while elderly age, uncommon EGFR-related mutations, and lymphocyte-to-monocyte ratio were found to be independent prognostic factors for OS. A useful prognostic scoring tool for improving the survival risk stratification of patients was established by incorporating the above essential factors. Baseline hypoalbuminemia and PLR could be crucial clinical assessment factors when initiating TKI therapy. In addition, the optimization of individualized treatment strategies for these patients may be assisted by using the risk-scoring model.

## 1. Introduction

Lung cancer is the leading cause of cancer-related deaths, with over 1.8 million deaths expected worldwide in 2021 [1]. Molecular targeted therapies and immunotherapies for non-small-cell lung cancer (NSCLC) have substantially enhanced clinical outcomes over previous decades. Among the targetable driver mutations, the epidermal growth factor receptor (EGFR) gene is the most common. Several effective tyrosine kinase inhibitors (TKIs) are now being used as a standard of care for EGFR-defined patients [2]. However, more than 70% of patients become resistant to targeted therapy and disease eventually progresses during treatment [3]. Drug resistance in patients with EGFR mutations cannot be precisely predicted. Moreover, reliable host factors are an essential and unmet requirement for obtaining predictive outcomes effectively [4].

In patients with NSCLC, cachexia and sarcopenia are commonly examined at the baseline. These symptoms are highly correlated with poor survival outcomes [5,6]. The degree to which sarcopenia is associated with treatment outcomes and survival of these patients remains unknown. Frailty is the state of increased vulnerability due to acute and chronic stressors. It is caused by a considerable reduction in physiologic reserves and increases the risk of unfavorable clinical outcomes [7]. A few studies showed that frailty is correlated with reduced overall survival in patients with lung cancer [8,9], and its evaluation can reveal a toxicity risk [10]. However, frailty’s predictive power and usefulness in patients with EGFR-mutated NSCLC have not yet been effectively demonstrated [11].

Extensive research has previously revealed that blood-based inflammatory/immune markers, including lymphocyte-to-monocyte ratio (LMR), neutrophil-to-lymphocyte ratio (NLR), platelet-to-lymphocyte ratio (PLR), and systemic immune-inflammation index (SII) are significant prognostic indicators of NSCLC [12,13,14,15]. These hematological markers can be detected inexpensively and conveniently in clinical practice and could possibly reflect tumor-promoting activities [16]. Unfortunately, status in all these biomarkers and their correlation with survival have not been established in advanced EGFR-mutated NSCLC.

So far, discovery of selective predictive factors for response or resistance to TKI in first-line therapy of patients with NSCLC has not been accomplished. Furthermore, the most common adverse events (AEs) of EGFR TKIs were diarrhea and rash. The prevalence and severity of other AEs, including interstitial lung disease (ILD) and liver function impairments that have been considered idiosyncratic and are usually not linked to the effectiveness of EGFR inhibition, are correlated with the potency of EGFR inhibition. The identification of patients who could benefit from specific predictive factors could thus facilitate tailoring of a personalized treatment. The present study aimed to comprehensively assess the characteristics and risk factors associated with TKI toxicity and explore the predictive power of systemic inflammation markers and clinical risk factors combined for the risk stratification of the survival of patients diagnosed with EGFR-mutated lung adenocarcinoma and receiving TKI therapy.

## 2. Materials and Methods

### 2.1. Patients and Study Design

This study was approved by the institutional review board of Hualien Tzu Chi Hospital, Buddhist Tzu Chi Medical Foundation, which waived the requirement for informed consent for de-identified data (IRB110-082-B). Patients who were newly diagnosed with EGFR-mutated lung adenocarcinoma from January 2008 to December 2020 at an academic medical center were retrospectively enrolled. All of the participants had pathologically diagnosed lung adenocarcinoma and had serial imaging studies for staging at initial diagnosis, including computed tomography (CT) of the chest to upper abdomen, whole-body bone scan, positron emission tomography/CT (PET/CT), and magnetic resonance imaging (MRI) of the brain. The patients were staged by the 7th edition of the staging manual by the American Joint Committee on Cancer (AJCC) [17]. The analysis included patients with an AJCC stage of IIIB or IV and an active EGFR mutation and presented available PET/CT images within 1 month before TKI administration. EGFR mutational analysis was conducted from the tumor specimen of histopathologically confirmed lung adenocarcinomas. The EGFR mutation examination was conducted on formalin-fixed, paraffin-embedded tissues of histologically verified lung adenocarcinoma. Mutation analyses were performed with a Therascreen EGFR RGQ PCR Kit and the Cobas^®^ EGFR mutation test version 2, which uses amplification refractory mutation-specific polymerase chain response and Scorpion technologies. All participants received EGFR-TKIs (gefitinib, erlotinib, or afatinib) as the first-line treatment. The TKI was selected on the basis of the decision of the clinical physician.

Baseline clinical features, including age at diagnosis, sex, smoking status, and alcohol history, Charlson comorbidity index (CCI), tumor characteristics, five-item modified frailty index (mFI-5), nutritional status, and treatment modalities were collected by an electronic chart review. CCI and mFI-5 were monitored as previously described [9,18]. Malignant pleural effusion and brain metastasis were detected via either pleural effusion cytology or a pleural biopsy and brain MRI. EGFR mutation analysis on tissue and complete blood count and albumin level determination on peripheral venous blood were performed simultaneously, and TKI treatment was started within 1 month. Pretreatment LMR, NLR, and PLR were assessed as the ratio of lymphocyte count to the absolute count of monocytes, neutrophil cell to lymphocyte cell count, and platelet count to lymphocyte cell count, respectively. SII was determined using the following equation: NLR × PLT.

### 2.2. Definition of Sarcopenia

Sarcopenia was determined on the basis of a single-slide CT measurement of the cross-sectional skeletal muscle (SMA) at the level of the third lumbar vertebra (L3) [19,20]. CT images from whole-body PET/CT scans were applied to evaluate SMA. SMA was quantified at the axial slice adjacent to the inferior aspect of the L3 by using a threshold within −29 to +150 Hounsfield units [19]. The region-growing algorithm [20] was utilized to facilitate the automatic segmentation of all skeletal muscle mass in the slice. Skeletal muscle contours on the CT image were modified when required. The L3 skeletal muscle index (cm^2^/m^2^) was calculated as SMA normalized by the square of the height. SMA images were analyzed with the open-source software OsiriX (Pixmeo, Geneva, Switzerland) [21]. Sarcopenia was specified by using the cutoff thresholds of <52.4 cm^2^/m^2^ for men and <38.5 cm^2^/m^2^ for women [22,23].

### 2.3. Toxicity Evaluation and Follow Up

Patients were followed up at the outpatient clinic at a 1-month interval. AEs and laboratory abnormalities were graded based on the National Cancer Institute Common Terminology Criteria for Adverse Events, version 5.0 [24]. Patients regularly received thoracic-to-abdominal contrast-enhanced CT every three months. Imaging survey and biopsy were performed when disease progression signs or symptoms were reported. TKI-treatment response was categorized based on chest CT or PET/CT imaging studies [25]. The time between the initiation of TKI therapy and the date of disease progression or the date of death or censoring of the last follow-up was defined as progression-free survival. The duration from the date of TKI treatment to the date of death or censoring of the last follow-up for surviving patients was considered as overall survival (OS).

### 2.4. Statistical Analyses

Mean, standard deviation, frequency, percentage, median, and interquartile range (IQR) were considered descriptive statistics. The correlation between clinical variables and TKI-related toxicity was assessed using logistic regression analysis. Survival curves were obtained via the Kaplan–Meier approach, and the log-rank test was used for comparison. OS and PFS were subjected to univariate and multivariate analyses by using the Cox proportional hazards model. The results of the analyses were presented as hazard ratio (HR) with 95% confidence interval (CI). The time-dependent ROC curve analyses of systemic inflammation indexes were performed with EZR (Saitama Medical Center, Jichi Medical University, Saitama, Japan), which is a graphical user interface for R (The R Foundation for Statistical Computing, Vienna, Austria). The prognostic scoring model for PFS and OS was established on the basis of independent features. Harrell’s C-index was applied to assess the prognostic performance of the models [26]. The model was validated by utilizing a bootstrapping approach for internal validation. Validation was conducted with 1000 bootstrap samples. The multivariable model with the highest c-index parameter was selected for the foundation of the prognostic factors. Statistics were analyzed via SPSS software version 25 (IBM, New York, NY, USA). A *p*-value < 0.05 was considered statistically significant.

## 3. Results

### 3.1. Baseline Characteristics

Among patients with advanced adenocarcinoma containing EGFR mutations that received TKIs between 1 January 2008 and 31 December 2020, 259 were retrospectively reviewed. A total of 316 patients with stage IIIB and IV lung adenocarcinoma and receiving TKI as the first-line treatment were included in the present study in accordance with the inclusion criteria. In the end, 259 patients were enrolled in the study by further referring to the exclusion criteria, and 49 patients without available images for SMI analysis before TKI therapy and eight patients who had incomplete or missing data were excluded. This cohort study involved 259 patients (123 men and 136 women) who met the inclusion criteria (Table 1). The median age was 71 years (IQR 62–78.7), while patients older than 70 years comprised 49% of the entire cohort. At the time of TKI treatment initiation, most patients had never smoked (63.7%), presented a high CCI risk (CCI ≥ 5, 63.3%), while 232 patients (89.6%) were of TNM stage IV. With respect to the EGFR mutation type, 115 (44.4%) with exon 19 deletions, 109 (42.0%) were confirmed with L858R mutation, and the remaining 35 (13.6%) had other uncommon mutation types. Approximately 60% of the patients had a normal weight and albumin level. The TKIs of gefitinib, afatinib, and erlotinib were used in 115 (44.4%), 79 (30.6%), and 65 (25%) patients, respectively. Most patients had no pleural effusion (64.5%) or brain metastasis (74.5%). Sarcopenia was identified in 159 patients (61.4%). The median period of first-line TKI administration was 10 months (IQR 3–18), while 144 of the patients (55.6%) did not receive any adjuvant therapy. In 18 patients, the response to first-line TKI treatment was stable disease. Meanwhile, the disease was progressive in 182 patients.

### 3.2. Characteristics Factors Associated with TKI-Related Adverse Events

AEs were evaluated in all enrolled patients (Table 2). Ten patients discontinued treatment because of grade 3 or 4 AEs, including grade 3 diarrhea reported in four patients, one had grade 4 hepatotoxicity, and five had acneiform rash, respectively. No ILD or treatment-related deaths were observed. The acneiform rash within the cohort was the most common AE, followed by diarrhea and anemia. Thirty patients experienced grade 3 AEs that were generally manageable. The main treatment-related toxicities of grades 3–4 were an increase in hepatic enzymes (11.5%), diarrhea (6%), and hypoalbuminemia (3.7%). Table 3 shows the logistic regression analysis of the baseline clinical features for the incidence of TKI-related toxicities. Limited objectives were reported, such as acneiform rash, anemia, hypoalbuminemia, and liver enzyme elevation. In multivariate analysis, independent predictors of incidence acneiform rash were lower albumin (odds ratio (OR), 0.445; 95% CI, 0.225–0.883). Baseline anemia (OR, 5.113; 95% CI, 3.372–7.851) and high PLR (OR, 2.122; 95% CI, 1.153–3.904) were associated with anemia incidence. Elderly age (OR, 2.594; 95% CI, 1.081–6.185), lower body mass index (BMI, OR, 6.801; 95% CI, 1.575–7.055), and lower albumin (OR, 9.348; 95% CI, 3.601–8.392) were higher risk factors of developed hypoalbuminemia.

### 3.3. Associated of Clinicopathological Factors on Survival Analysis

The median follow-up time from the date of diagnosis was 100 months (range, 6–173 months). The OS and PFS were 32.5% and 16.8%, respectively. The cutoff values of LMR, NLR, PLR, and SII as factors of the OS and PFS are presented in Supplementary Table S1 and Figure S1, respectively. Univariate and multivariate Cox regression analyses were used to predict PFS and OS (Table 4).

The univariate analysis model was used to fit the significant inflammatory indexes and clinical features. Then, multivariate analysis was performed. After multivariate analysis, elderly age, stage IV, mutations including L858R type and other uncommon types, as well as higher NLR were independent risk factors for lower PFS. Elderly age, uncommon mutation types, and higher LMR were significantly associated with OS. Supplementary Figure S2 presents the Kaplan–Meier survival plots that were generated on the basis of independent prognostic features.

### 3.4. Development of Prognostic Scoring Model

All significantly prognostic features for PFS and OS were included after 1000 bootstrap replications. The remarkable similarity between the regression parameters acquired from 1000 bootstrap samples and those obtained from the original Cox model was suggestive of successful internal validation (Table 5). Risk factors involved age and stage status among clinical variables, NLR and LMR among hematological indexes, and exon deletion among mutation types. In accordance with the bootstrapped Cox model coefficients, points were assigned by using the regression coefficient based on the Schneeweiss scoring system [27]. These points varied from a minimum of 0 to a maximum of 8 and 13 scores for PFS and OS, respectively.

**Figure 1:**
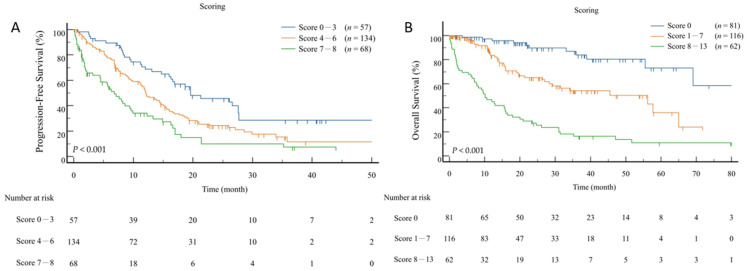
Kaplan–Meier estimates of progression-free survival (**A**) and overall survival (**B**) according to the prognostic scoring model.

Regarding PFS, the eight prognostic features were added in three prognostic groups characterized as follows: prognostic group 1 (score 0–3), prognostic group 2 (score 4–6), and prognostic group 3 (score 7–8) (Figure 1A). According to the PFS score, prognostic group 1 (*n* = 57, 22%) presented a significantly higher PFS (38 months) compared to prognostic group 2 (*n* = 134, 52%, PFS = 18 months, HR, 1.742, *p* = 0.005) and prognostic group 3 (*n* = 68, 26%, PFS = 10 months, HR, 2.965, *p* < 0.001). With respect to OS in contrast (Figure 1B), the thirteen prognostic features were added in three prognostic groups characterized as follows: prognostic group 1 (score 0), prognostic group 2 (score 1–7), and prognostic group 3 (score 8–13). Based on OS score, prognostic group 1 (*n* = 81, 31%) demonstrated a significantly higher OS (72 months) than prognostic group 2 (*n* = 116, 45%, OS = 42 months, HR, 3.328, *p* < 0.001), and prognostic group 3 (*n* = 62, 24%, OS = 21 months, HR, 10.377, *p* < 0.001).

## 4. Discussion

Approximately 70% of lung adenocarcinoma patients harboring EGFR-mutated develop resistance against TKI therapy, and cancer eventually progresses during treatment within the next year [3]. In addition, up to 30% of patients treated with TKI need dose conversions because of AEs [2]. A more accurate prediction factor and approach is urgently required for improved safety and efficacy of TKI treatment. Based on the literature review, the current study accesses the systemic inflammation indices and clinical features associated with the TKI treatment-related toxicities and survival outcomes of EGFR-mutated lung adenocarcinoma patients receiving first-line TKI therapy. Within the cohort, it was discovered that during the initial TKI therapy, hypoalbuminemia was significantly correlated with acneiform rash and post-TKI hypoalbuminemia. In addition, anemia and higher PLR were significantly correlated with post-TKI anemia. Furthermore, it was discovered that higher NLR, elderly age, stage IV, mutation of L858R, and uncommon type were independent risk factors of PFS, while lower LMR, elderly age, and mutation of uncommon type were independent risk factors of OS.

It was also discovered that hypoalbuminemia before treatment was significantly correlated with acneiform rash after treatment. Moreover, elderly age and lower BMI were independent risk factors for clinical variables in post-treatment hypoalbuminemia. TKIs are highly protein-bound therapeutics, and the occurrence of hypoalbuminemia may consequence in enhanced free-drug concentrations, directing thus increased drug exposure and AEs [28,29]. Albumin and BMI are commonly employed as markers of nutritional conditions, may be considered as inflammation parameters [30,31], and are related to age and cancer disease status [32]. Subsequently, TKI-related AEs such as diarrhea might lead to poor nutritional status. In addition, some patients might also have experienced deterioration in nutritional status after treatment. Baseline hypoalbuminemia and anemia could be critical clinical examinations when starting TKIs, but further investigations are required to demonstrate an association between these two variables and TKI-related AEs. PLR is an inflammation marker and could be responsible for reduced hemoglobin levels. Thrombocytosis was correlated with acute blood loss, chronic inflammation, infection disease, and iron deficiency anemia [33]. Nevertheless, further research is required to demonstrate a correlation between higher PLR and anemia during TKI therapy.

The incidence of grade 3 acneiform rash in the study’s cohort (2.8%) is in accordance with previously published frequency [34]. Importantly, incidence of liver toxicity in the present study appears to be higher. In accordance with earlier findings, incidence of liver toxicity is higher in Asian patients compared to non-Asian patients [35]. The abovementioned AEs can influence the patients’ quality of life and can often lead to a termination of the anticancer therapy [36]. The previous study has shown that sarcopenia is correlated with treatment-related toxicity [37] and survival [38] in various malignancies. Nevertheless, the present study has not observed a correlation between treatment outcome and sarcopenia or frailty status. With respect to the correlation between sarcopenia and prognosis, previous studies have not revealed differences in postoperative AEs in patients with lung cancer with or without sarcopenia [39,40,41]. It has not yet been demonstrated whether sarcopenia could affect the response of AEs to TKIs or survival in NSCLC [11]. Minami et al. [42] collected 167 patients with NSCLC and EGFR mutations who received TKI therapy and found no difference in survival results in accordance with sarcopenia status. Rossi et al. [43] reported no difference between the incidence rates of sarcopenia or non-sarcopenia AE in patients with NSCLC and the EGFR mutation. However, these two previous studies had a limited sample size. Moreover, as recommended by the updated Asian Working Group for Sarcopenia [44], the measurement of muscle mass independence does not account for the impairment of muscle function in sarcopenia. The practical measure of muscle power and physical performance should be performed on patients with sarcopenia. The association between sarcopenia and treatment-related toxicity and survival in patients with lung adenocarcinoma and EGFR mutations needs further research. The frailty findings in our study may be influenced by comorbidities because the combined risk of major comorbidities in our population was not assessed by using the mFI-5 assessment tool. Additionally, frailty assessment is insufficient in patients with NSCLC receiving TKI treatment and immunotherapy [11,45]. The association between sarcopenia or frailty status and TKI-related toxicity in NSCLC, thus, requires additional research in the future.

The results of the present study are in accordance with findings showing that age, late-stage disease (stage IV), EGFR mutation types, and systemic inflammatory biomarkers are correlated with clinical outcomes prognosis [46,47,48]. These results supplement the increasing evidence of risk factors for EGFR-mutant NSCLC treated with TKIs. The present findings can produce a predictive scoring system that lets physicians acquire an exhaustive report on managing the therapeutic strategy for EGFR-mutated lung adenocarcinoma. These parameters should help the survival prediction and management of patients. The combination of LMR, mutation of uncommon types (S768I in exon 20, G719X in exon 18, and two-point mutations with E709G/L858R and G719X/L861Q), and ages of at least 70 years old can pave the way for the construction of a risk-scoring system and the improvement of OS stratification. A PFS risk-scoring system integration for ages of at least 70 years old, stage IV, mutations including L858R and uncommon types, and NLR were correlated with reduced PFS. A significant proportion of NSCLC patients with EGFR mutations show resistance to EGFR TKIs with short-term PFS [47]. In addition, a growing body of evidence has demonstrated that the outcomes of patients with unusual EGFR mutations, such as G719X and L861Q, and complex mutations improve upon treatment with second-generation TKIs [48,49]. The combination of therapeutic strategies with immune modulation has also been proposed and presented efficient responses in patients harboring EGFR mutations [50]. The T790M mutation in EGFR exon 20 accounts for almost half of cases and is the most common type of acquired resistance [51]. However, our cohort retrospective study lacked repetition examined on the EGFR T790M mutation. The third-generation TKI osimertinib was devised to control T790M mutation-induced resistance [52]. In prospective studies, the T790M mutation should be incorporated into our scoring model.

Elucidation of the mechanisms underlying low LMR and high NLR effects on the oncologic outcomes remains unclear. Previous evidence supported that systemic immune and inflammatory cells affect multiple pathways and, thus, have essential roles in tumor initiation, proliferation, invasion, and migration [53,54,55]. Moreover, the prognostic role of LMR in lung cancer has been previously confirmed [56]. As markers of the antitumor immune response, lymphocytes, particularly tumor-infiltrating lymphocytes, play a crucial function in causing cytotoxic cell death and hindering tumor expansion and migration by stimulating cytokines [54]. Monocytes are drafted to tumor sites, determined to be tumor-related macrophages, and polarized to M2 macrophages with inferior antigen-presenting potential and Th1-adaptive resistance [57]. Decreased lymphocyte counts are thus considered to be responsible for an incompetent tumor’s immunologic reaction [56]. Furthermore, neutrophils may instantly impair the extracellular matrix to stimulate tumor cell aggression and inhibit the cytolytic action of lymphocytes and other immune cells [58]. LMR and NLR are potential clinical biomarkers that are easy to determine, can be repeatedly obtained and are of low-cost. Based on the above results, the proposed scoring model could identify other risk groups of patients eligible for novel therapeutic strategies that are expected to become a promising predictor, guiding the individualized TKIs of NSCLC with EGFR mutation.

Despite the present study’s analysis involving a significantly larger patient cohort, it can be concluded that the current study is exploratory and contains certain limitations. Initially, as a retrospective study, the outcome cannot be considered as definitive. Additional extensive cohort studies need to be conducted to specify the cutoff threshold of continuous biomarkers. Finally, although an internal validation of the scoring system was conducted in the present study, generalizations of the study’s findings require future large prospective cohort studies.

## 5. Conclusions

Age was significantly correlated with TKI-related hypoalbuminemia and survival outcomes. Furthermore, LMR, NLR, and mutation types can be used as independent prognostic markers of survival in patients diagnosed with lung adenocarcinoma harboring EGFR mutations receiving TKIs. Incorporating age, inflammatory indexes, mutation type, and tumor stage (only for PFS) represents a useful prognostic scoring tool for enhancing the risk stratification of patients. This risk-scoring model should support optimizing individualized therapy strategies for these patients. 

## Figures and Tables

**Table 1:** Patient characteristics (*n* = 259).

Variable	*n* (%)
Age, median (IQR)	71.0 (62.0–78.7)
<70/≥70	133 (51.4)/126 (48.6)
Sex (Male/Female)	123 (47.5)/136 (52.5)
Smoking	
Never/Former/Current	165 (63.7)/14 (5.4)/80 (30.9)
CCI, median (IQR)	6 (2.0–8.0)
<5/≥5	95 (36.7)/164 (63.3)
mFI-5, median (IQR)	1 (0–2)
0/1/≥2	87 (33.6)/82 (31.7)/90 (34.7)
Cancer stage (IIIB/IV)	27 (10.4)/232 (89.6)
Mutation type of EGFR	
Deletion 19/L858R/others	115 (44.4)/109 (42.0)/35 (13.6)
Frist line TKIs	
Afatinib/Erlotinib/Gefitinib	79 (30.6)/65 (25.0)/115 (44.4)
Pleural effusion (No/Yes)	167 (64.5)/92 (35.5)
Brain metastasis (No/Yes)	193 (74.5)/66 (25.5)
BMI, kg/m^2^, mean (SD)	22.9 (3.7)
<18.5/18.5–24.9/≥25.0	27 (10.4)/157 (60.6)/75 (29)
Albumin, g/L, median (IQR)	3.6 (3.1–4.1)
≥3.5/<3.5	102 (60)/68 (40)
Hemoglobin, g/dL, mean (SD)	12.5 (1.8)
≥11/<11	209 (80.7)/50 (19.3)
Sarcopenia (No/Yes)	100 (38.6)/159 (61.4)
SMI, cm^2^/m^2^, median (IQR)	42.8 (37.2–50.0)
Sarcopenia (Male/Female)	86 (54)/73 (46)
Time of TKIs treatment, median (IQR), month	10 (3–18)
Adjuvant therapy	
None/Radiotherapy	144 (55.6)/15 (5.8)
Chemotherapy/Concurrent chemoradiotherapy	91 (35.1)/9 (3.5)

BMI: body mass index; CCI: Charlson comorbidity index; EGFR: epidermal growth factor receptor; LMR: lymphocyte-to-monocyte ratio; IQR: interquartile range; mFI-5: five-item modified frailty index; NLR: neutrophil-to-lymphocyte ratio; PLR: platelet-to-lymphocyte ratio; SD: standard deviation; SII: systemic immune inflammation index; SMI: skeletal muscle index; TKIs: tyrosine kinase inhibitors.

**Table 2:** Adverse events during treatment with tyrosine kinase inhibitors (*n* = 259).

Adverse Event	Total	Grade 1	Grade 2	Grade 3	Grade 4	Grade 3–4
Acneiform rash	176 (68)	145 (82.4)	26 (14.8)	5 (2.8)	0 (0)	5 (2.8)
Diarrhea	117 (45.2)	97 (82.9)	13 (11.1)	7 (6)	0 (0)	7 (6)
Anemia	100 (38.6)	76 (76)	21 (21)	3 (3)	0 (0)	3 (3)
Hypoalbuminemia	80 (30.9)	43 (53.8)	34 (42.5)	3 (3.7)	0 (0)	3 (3.7)
Neutropenia	12 (4.6)	11 (91.7)	1 (8.3)	0 (0)	0 (0)	0 (0)
AST increase	61 (23.6)	51 (83.6)	3 (4.9)	6 (9.9)	1 (1.6)	7 (11.5)
ALT increase	61 (23.6)	51 (83.6)	3 (4.9)	6 (9.9)	1 (1.6)	7 (11.5)

ALT: alanine aminotransferase; AST: aspartate aminotransferase.

**Table 3:** Univariate and multivariate logistic regression analyses of clinical variables in TKI-related toxicity outcomes.

Variable	Acneiform Rash			Anemia			Hypoalbuminemia			Liver Enzyme Elevation
	Univariate	Multivariate		Univariate	Multivariate		Univariate	Multivariate		Univariate
	*p*	HR (95% CI)	*p*	*p*	HR (95% CI)	*p*	*p*	HR (95% CI)	*p*	*p*
Age (<70 vs. ≥70)	0.027	0.671 (0.331–1.32)	0.249	0.060			0.004	2.594 (1.081–6.185)	0.031	0.127
CCI (<5 vs. ≥5)	0.176			0.673			0.776			0.068
mFI-5 (0 vs. 1 vs. ≥2)	0.592			0.785			0.969			0.626
Cancer stage (IIIB vs. IV)	0.023	0.942 (0.423–2.111)	0.880	0.604			0.631			0.283
Pleural effusion (no vs. yes)	0.796			0.045	0.612 (0.334–1.131)	0.119	0.034	1.517 (0.712–4.263)	0.218	0.186
Sarcopenia (no vs. yes)	0.226			0.024	1.773 (0.961–3.281)	0.067	0.396			0.079
BMI, kg/m^2^ (18.5–24.9 vs. <18.5 vs. ≥25)	0.121			0.113			0.024	6.801 (1.575–7.055)	0.009	0.452
Albumin, g/L (≥3.5 vs. ≤3.5)	0.007	0.445 (0.225–0.883)	0.020	0.065			<0.001	9.348 (3.601–8.392)	<0.001	0.048
Hemoglobin, g/L (≥11 vs. <11)	0.831			<0.001	5.113 (3.372–7.851)	<0.001	0.364			0.449
LMR (continuous variable)	0.319			0.025	0.801 (0.342–1.881)	0.621	0.280			0.695
NLR (continuous variable)	0.412			0.168			0.011	1.117 (0.395–3.516)	0.774	0.991
PLR (continuous variable)	0.631			0.006	2.122 (1.153–3.904)	0.015	0.251			0.673
SII (continuous variable)	0.593			0.026	1.321 (0.614–2.846)	0.472	0.417			0.067

BMI: body mass index; CCI: Charlson comorbidity index; CI: Confidence interval; HR, hazard ratio; LMR: lymphocyte-to-monocyte ratio; mFI-5: five-item modified frailty index; NLR: neutrophil-to-lymphocyte ratio; PLR: platelet-to-lymphocyte ratio; SII: systemic immune inflammation index.

**Table 4:** Univariate and multivariate Cox regression analyses for the prediction of survival outcomes.

Variable	Progression-Free Survival				Overall Survival			
	Univariate		Multivariate		Univariate		Multivariate	
	HR (95% CI)	*p*	HR (95% CI)	*p*	HR (95% CI)	*p*	HR (95% CI)	*p*
Age (<70 vs. ≥70)	1.330 (1.001–1.790)	0.048	1.47 (1.085–2.001)	0.013	1.985 (1.341–2.931)	<0.001	1.891 (1.182–3.011)	0.008
Sex (male vs. female)	1.221 (0.912–1.634)	0.176			1.171 (0.762–1.731)	0.412		
Smoking (never vs. former vs. current)	1.371 (0.751–2.506)	0.298			2.122 (1.054–4.311)	0.036	1.452 (0.635–3.303)	0.373
CCI (<5 vs. ≥5)	1.152 (0.854–1.563)	0.357			0.561 (0.382–0.820)	0.003	0.824 (0.511–1.334)	0.440
mFI-5 (0 vs. 1 vs. ≥2)	1.161 (0.822–1.655)	0.381			1.920 (1.181–3.156)	0.009	1.041 (0.591–1.852)	0.879
Cancer stage (IIIB vs. IV)	2.203 (1.221–3.963)	0.008	1.91 (1.021–3.571)	0.043	2.140 (0.991–4.635)	0.050		
EGFR Mutation (deletion 19 vs. L858R)	1.464 (1.071–1.992)	0.010	1.50 (1.085–2.102)	0.015	1.271 (0.840–1.952)	0.252		
EGFR Mutation (deletion 19 vs. others)	1.925 (1.181–3.133)	0.008	2.36 (1.424–3.910)	<0.001	2.902 (1.622–5.204)	<0.001	3.072 (1.57–5.99)	<0.001
Pleural effusion (no vs. yes)	1.262 (0.941–1.712)	0.119			1.323 (0.885–1.961)	0.171		
Brain metastasis (no vs. yes)	1.141 (0.823–1.582)	0.420			1.094 (0.712–1.691)	0.667		
Sarcopenia (no vs. yes)	1.024 (0.765–1.394)	0.850			1.612 (1.053–2.460)	0.028	1.361 (0.865–2.156)	0.179
BMI, kg/m^2^ (18.5–24.9 vs. <18.5 vs. ≥25)	1.145 (0.821–1.582)	0.420			1.565 (0.974–1.672)	0.059	1.656 (0.741–3.663)	0.215
Albumin, g/L (≥3.5 vs. ≤3.5)	1.474 (1.085–1.963)	0.010	1.101 (0.620–1.681)	0.569	1.301 (0.866–1.901)	0.144		
LMR (high vs. low)	1.481 (1.096–1.993)	0.009	1.121 (0.741–1.712)	0.567	9.963 (5.740–11.303)	<0.001	5.371 (2.451–9.751)	<0.001
NLR (low vs. high)	1.841 (1.271–2.662)	0.001	1.742 (1.191–2.556)	0.004	3.241 (1.763–5.941)	<0.001	1.575 (0.642–3.844)	0.316
PLR (low vs. high)	1.533 (1.135–2.071)	0.005	1.231 (0.910–1.700)	0.185	6.753 (4.086–8.155)	<0.001	2.024 (0.945–4.315)	0.069
SII (low vs. high)	1.661 (1.131–2.463)	0.009	0.903 (0.513–1.532)	0.706	2.804 (1.635–4.801)	<0.001	0.653 (0.256–1.650)	0.371

BMI: body mass index; CCI: Charlson comorbidity index; CI: confidence interval; EGFR: epidermal growth factor receptor; HR: hazard ratio; LMR: lymphocyte-to-monocyte ratio; mFI-5: five-item modified frailty index; NLR: neutrophil-to-lymphocyte ratio; PLR: platelet-to-lymphocyte ratio; SII: systemic immune inflammation index.

**Table 5:** Multivariate Cox regression coefficients and prognostic scoring definition.

Variable			Bootstrap (1000 Replication)					Bootstrap (1000 Replication)		
	Original Dataset (*n* = 259)		Progression-Free Survival			Original Dataset (*n* = 259)		Overall Survival		
	β-Coefficient ± SE	*p*	β-Coefficient ± SE	*p*	Score	β-Coefficient ± SE	*p*	β-Coefficient ± SE	*p*	Score
Age (<70 vs. ≥70)	0.377 ± 0.161	0.013	0.379 ± 0.156	0.015	1	0.649 ± 0.242	0.242	0.604 ± 0.221	0.006	2
EGFR Mutation (deletion 19 vs. L858R)	0.409 ± 0.176	0.002	0.409 ± 0.164	0.013	1					
EGFR Mutation (deletion 19 vs. others)	0.814 ± 0.299	0.030	0.817 ± 0.256	0.001	3	1.183 ± 0.415	0.415	1.162 ± 0.319	<0.001	4
Cancer stage (IIIB vs. IV)	0.713 ± 0.369	0.016	0.715 ± 0.318	0.024	2					
LMR (high vs. low)						2.196 ± 0.289	0.289	2.218 ± 0.283	<0.001	7
NLR (low vs. high)	0.534 ± 0.183	0.007	0.536 ± 0.190	0.005	2					

EGFR: epidermal growth factor receptor; LMR: lymphocyte-to-monocyte ratio; NLR: neutrophil-to-lymphocyte ratio; SE: standard error.

## Data Availability

The data presented in this study are available on request from the corresponding author. The data are not publicly available due to the privacy and ethical restrictions.

## Supplementary Materials

The following supporting information can be downloaded at: https://www.mdpi.com/article/10.3390/jpm12030404/s1, Table S1: Time-dependent receiver operating characteristic curve analysis for progression-free and overall survival; Figure S1: Time-dependent receiver operating characteristic curves for determining the optimal cutoff value of preoperative systemic inflammatory markers for progression-free survival included lymphocyte-to-monocyte ratio (A), neutrophil-to-lymphocyte ratio (B), platelet-to-lymphocyte ratio (C), and systemic immune-inflammation index (D); overall survival included lymphocyte-to-monocyte ratio (A), neutrophil-to-lymphocyte ratio (B), platelet-to-lymphocyte ratio (C), and systemic immune-inflammation index (D); Figure S2: Kaplan–Meier estimates of survival outcomes. Prediction of progression-free survival according to age (A), stage (B), EGFR-mutation type of deletion 19 vs. L858R (C), deletion 19 vs. others (D), and neutrophil-to-lymphocyte ratio (E). Prediction of overall survival according to age (F), EGFR-mutation type of deletion 19 vs. others (G), and lymphocyte-to-monocyte ratio (H).
