# Supplementary material for: Prognostic Values of Inflammatory Indexes and Clinical Factors in Patients with Epidermal Growth Factor Receptor Mutations in Lung Adenocarcinoma and Treated with Tyrosine Kinase Inhibitors
Source: J Pers Med. 2022 Mar 5;12(3):404. doi: 10.3390/jpm12030404 (PMC8955131; doi:10.3390/jpm12030404)
Supplement: Supplementary file 1 [file jpm-12-00404-s001.zip › jpm-1586715-supplementary.pdf]

Supplementary Online Content

Chang B-S, Peng T-C, Wu Y-F, Hsieh Y-C and Huang C-H. Prognostic Values of Inflammatory Indexes and Clinical Factors in Patients with Epidermal Growth Factor Receptor Mutations in Lung Adenocarcinoma and Treated with Tyrosine Kinase Inhibitors. *Journal of Personalized Medicine.*

Table S1. Time-dependent receiver operating characteristic curve analysis for progression-free and overall survival.

Figure S1. Time-dependent receiver operating characteristic curves for determining the optimal cutoff value of preoperative systemic inflammatory markers for progression-free survival included lymphocyte-to-monocyte ratio (**A**), neutrophil-to-lymphocyte ratio (**B**), platelet-to-lymphocyte ratio (**C**), and systemic immune-inflammation index (**D**); overall survival included lymphocyte-to-monocyte ratio (**A**), neutrophil-to-lymphocyte ratio (**B**), platelet-to-lymphocyte ratio (**C**), and systemic immune-inflammation index (**D**).

Figure S2. Kaplan–Meier estimates of survival outcomes. Prediction of progression-free survival according to age (**A**), stage (**B**), EGFR-mutation type of deletion 19 vs. L858R (**C**), deletion 19 vs. others (**D**), and neutrophil-to-lymphocyte ratio (**E**). Prediction of overall survival according to age (**F**), EGFR-mutation type of deletion 19 vs. others (**G**), and lymphocyte-to-monocyte ratio (**H**).

This supplementary material has been provided by the authors to give readers additional information about their work.

Table S1. Time-dependent receiver operating characteristic curve analysis for progression-free and overall survival.

	Progression-Free Survival				Overall Survival			
**Parameter**	**LMR**	**NLR**	**PLR**	**SII**	**LMR**	**NLR**	**PLR**	**SII**
AUC	0.547	0.643	0.550	0.562	0.873	0.579	0.793	0.617
Optimal sensitivity (%)	0.271	0.694	0.261	0.821	0.702	0.824	0.641	0.758
Optimal specificity (%)	0.816	0.599	0.913	0.374	0.696	0.361	0.844	0.496
Cutoff value at maximizes the sum of sensitivity and specificity	2.1	3.0	170.2	567.7	2.9	3.2	190.4	753.8

AUC: area under curve; LMR: lymphocyte-to-monocyte ratio; NLR: neutrophil-to-lymphocyte ratio; PLR: platelet-to-lymphocyte ratio; SII: systemic immune-inflammation index.

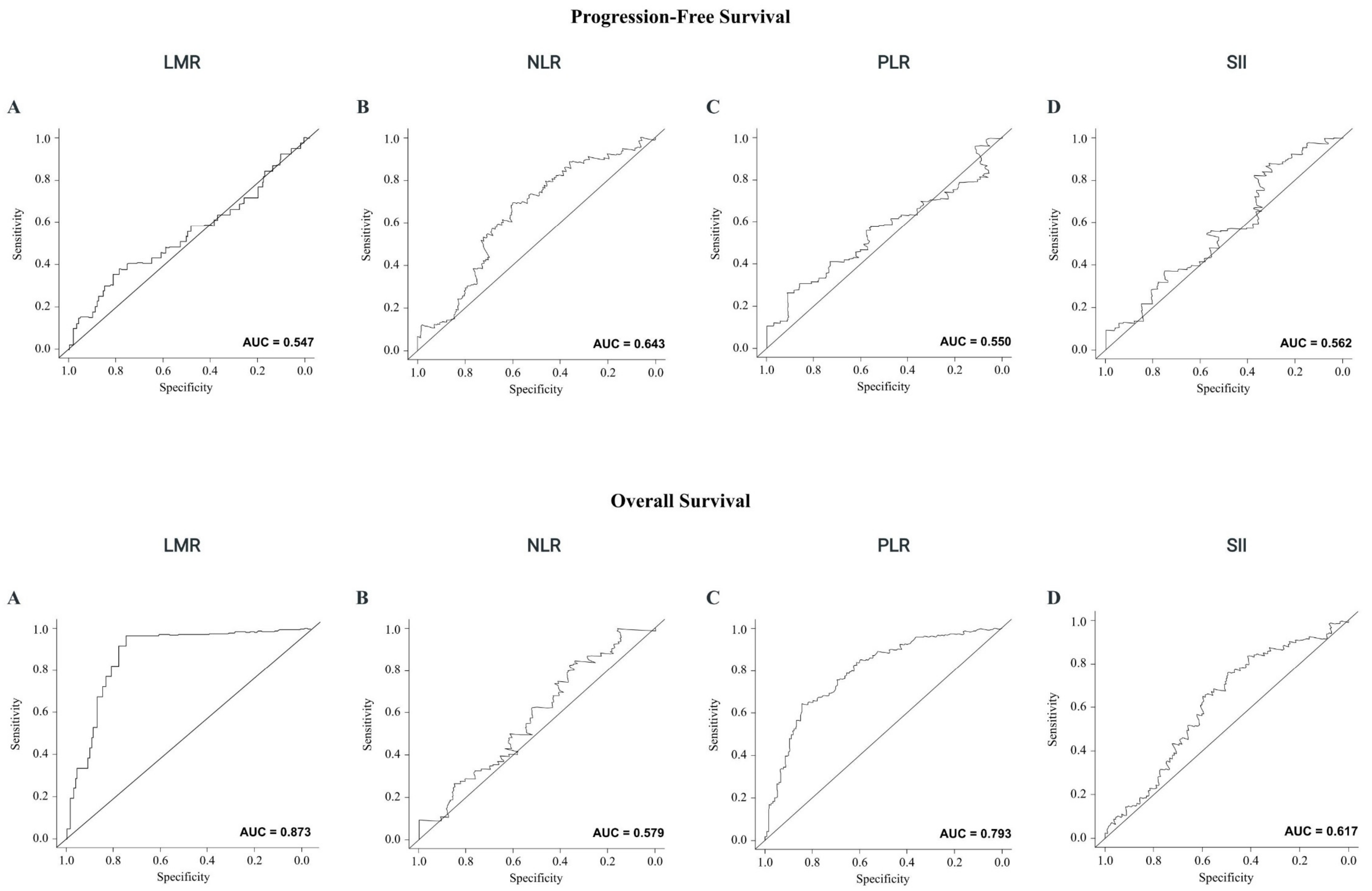

Figure S1. Time-dependent receiver operating characteristic curves for determining the optimal cutoff value of preoperative systemic inflammatory markers for progression-free survival included lymphocyte-to-monocyte ratio (**A**), neutrophil-to-lymphocyte ratio (**B**), platelet-to-lymphocyte ratio (**C**), and systemic immune-inflammation index (**D**); overall survival included lymphocyte-to-monocyte ratio (**A**), neutrophil-to-lymphocyte ratio (**B**), platelet-to-lymphocyte ratio (**C**), and systemic immune-inflammation index (**D**).

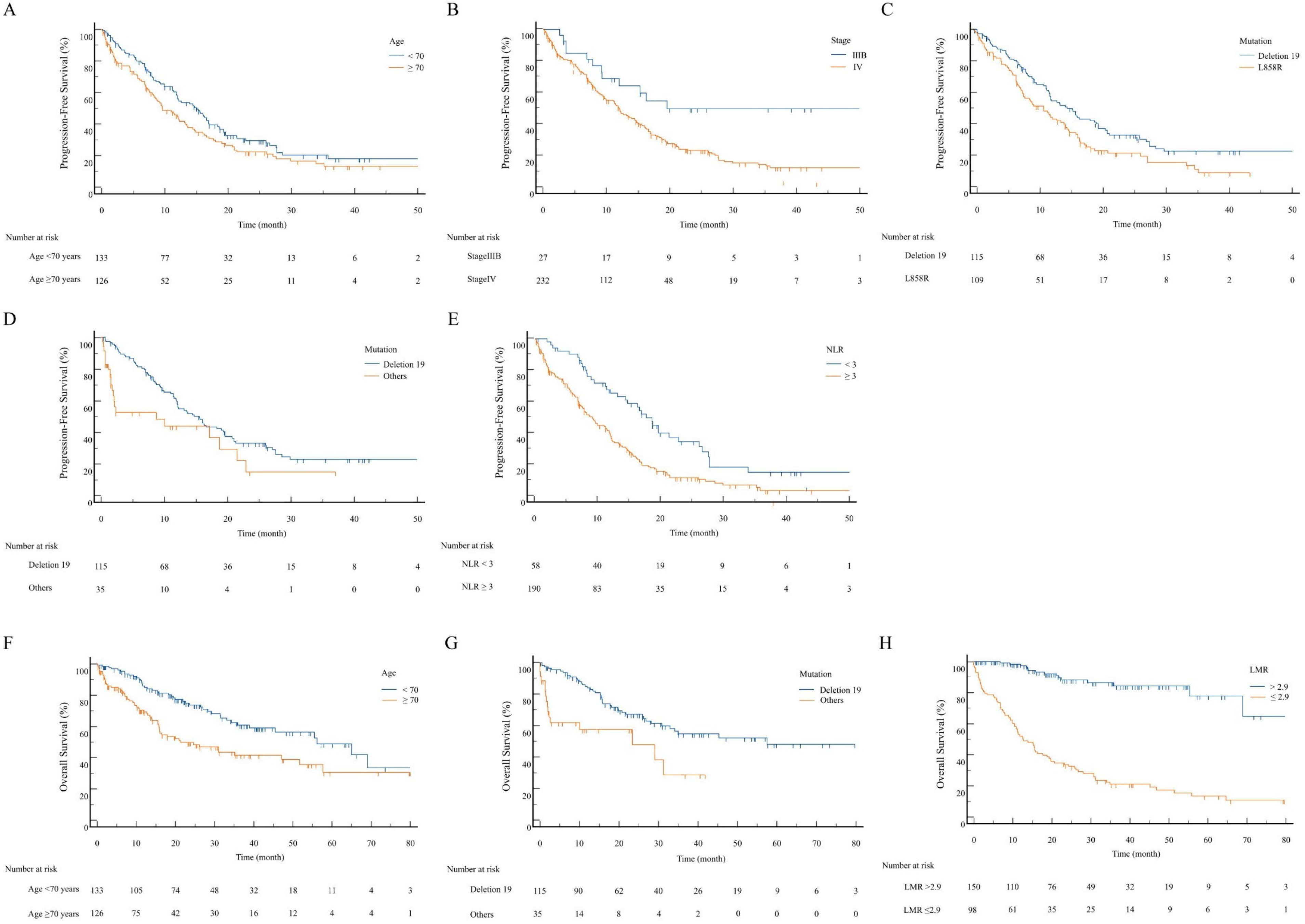

Figure S2. Kaplan–Meier estimates of survival outcomes. Prediction of progression-free survival according to age (**A**), stage (**B**), EGFR-mutation type of deletion 19 vs. L858R (**C**), deletion 19 vs. others (**D**), and neutrophil-to-lymphocyte ratio (**E**). Prediction of overall survival according to age (**F**), EGFR-mutation type of deletion 19 vs. others (**G**), and lymphocyte-to-monocyte ratio (**H**).
